# Comparison between two packages for pectoral muscle removal on mammographic images

**DOI:** 10.1007/s11547-022-01521-5

**Published:** 2022-07-11

**Authors:** Mario Sansone, Stefano Marrone, Giusi Di Salvio, Maria Paola Belfiore, Gianluca Gatta, Roberta Fusco, Laura Vanore, Chiara Zuiani, Francesca Grassi, Maria Teresa Vietri, Vincenza Granata, Roberto Grassi

**Affiliations:** 1 Department of Electrical Engineering and Information Technologies, University “Federico II”, Naples, Italy; 2 Department of Precision Medicine Division of Radiology, University of Campania Luigi Vanvitelli, Naples, Italy; 3 Medical Oncology Division, Igea SpA, Naples, Italy; 4 Institute for Diagnostic Radiology, University of Udine, Udine, Italy; 5 Department of Precision Medicine, Molecular and Clinical Pathology Unit, University of Campania Luigi Vanvitelli, Naples, Italy; 6 Division of Radiology, “Istituto Nazionale Tumori IRCCS Fondazione Pascale - IRCCS Di Napoli”, Naples, Italy; 7 Italian Society of Medical and Interventional Radiology (SIRM), SIRM Foundation, via della Signora 2, 20122 Milan, Italy

**Keywords:** Breast evaluation, Pectoral muscle removal, Full-field digital mammography

## Abstract

**Background:**

Pectoral muscle removal is a fundamental preliminary step in computer-aided diagnosis systems for full-field digital mammography (FFDM). Currently, two open-source publicly available packages (LIBRA and OpenBreast) provide algorithms for pectoral muscle removal within Matlab environment.

**Purpose:**

To compare performance of the two packages on a single database of FFDM images.

**Methods:**

Only mediolateral oblique (MLO) FFDM was considered because of large presence of pectoral muscle on this type of projection. For obtaining ground truth, pectoral muscle has been manually segmented by two radiologists in consensus. Both LIBRA’s and OpenBreast’s removal performance with respect to ground truth were compared using Dice similarity coefficient and Cohen-kappa reliability coefficient; Wilcoxon signed-rank test has been used for assessing differences in performances; Kruskal–Wallis test has been used to verify possible dependence of the performance from the breast density or image laterality.

**Results:**

FFDMs from 168 consecutive women at our institution have been included in the study. Both LIBRA’s Dice-index and Cohen-kappa were significantly higher than OpenBreast (Wilcoxon signed-rank test *P* < 0.05). No dependence on breast density or laterality has been found (Kruskal–Wallis test *P* > 0.05). Conclusion: Libra has a better performance than OpenBreast in pectoral muscle delineation so that, although our study has not a direct clinical application, these results are useful in the choice of packages for the development of complex systems for computer-aided breast evaluation.

## Introduction

Worldwide, breast cancer is the second most commonly diagnosed cancer, with approximately 2.1 million new diagnoses and almost 627,000 breast cancer-related deaths estimated to have occurred in 2018 [1, 2]. Breast cancer is a biologically and clinically heterogeneous disease, with several accepted histotypes, profiles of risk factors, responses to treatments and prognoses [1, 2]. The risk of developing breast cancer varies among women. Genetic susceptibility, factors affecting levels of endogenous hormones or exogenous hormone intake, lifestyle patterns, anthropometric characteristics, a high mammographic breast density and benign breast diseases are all associated with an increased risk of breast cancer [1, 2].

Screening mammography, with full-filed digital mammography (FFDM), is the method most commonly used worldwide for the detection of early tumor in asymptomatic women, and it is the only imaging modality proven to significantly lower cancer-related mortality [3]. Today, mammograms are evaluated by dedicated radiologists; however, during screening program, the high number of examinations to be evaluated and the complexity of the mammography features to be analyzed are elements that can influence the accuracy of the test. So, in this context, it has generated the necessity for systems for computer-aided diagnosis (CAD) to support radiologists in breast cancer detection and diagnosis [4–6].

Mammogram preprocessing is one of the primary steps in a CAD system. In the preprocessing step, the unnecessary elements are removed from the mammograms, which include annotations, labels, and background noises. The preprocessing allows the localization of region for abnormality search. In mammogram preprocessing, one of the major challenges is to accurately define the pectoral muscle (PM) boundary from the rest of the breast region. In fact, pixel intensities from pectoral muscle can be quite similar to masses and breast tissues and can affect lesion detection [7–9].

Several approaches have been proposed in the literature for pectoral muscle removal. Mainly, these algorithms have been based upon some kind of thresholding (adaptive histogram, region-growing) together with straight/curve line detection (Hough transform, Radon transform, wavelet transform, Euclidean regression) [8, 9, 16, 17, 24, 25]. Due to the specific appearance of pectoral muscle on MLO images (usually the pectoral muscle appears within MLO mammograms as a triangular region), the most spread approaches tried to exploit some a priori anatomical information and have been based on the Hough transform, an algorithm capable of detecting straight lines onto an image [7–9]. However, none of the above mentioned algorithms achieved optimal performance because of the precise shape of the pectoral border that is often curved (not straight) and because of the intensity levels which, as mentioned, often superimpose over the breast tissue intensities. Moreover, pectoral muscles are not always clearly visible in mammograms, especially of dense breasts, due to a poor contrast between pectoral muscle and breast parenchyma. Therefore, research is still active in this area. When developing complex pipelines for CAD breast evaluation or mass detection, researchers often should address the pectoral muscle removal as a first task; the availability of off-the-shelf tools for this task might greatly improve and simplify the overall design of a CAD system.

In this context, the Laboratory for Individualized Breast Radiodensity Assessment (LIBRA) [8] and OpenBreast [9] are two open-source packages available in Matlab environment (The MathWorks, Inc., Natik, MA, USA) [10]. In particular, these tools have been specifically developed for breast density evaluation, including pectoral muscle removal as a preprocessing step. However, each package performance has been evaluated on proprietary database of images acquired on different machines with different parameters. As a consequence, performance is not directly comparable since the different characteristics of the images might affect the results. Therefore, it could be interesting to evaluate the performance of available tools on a common database.

The primary endpoint of this study is to compare the performance of LIBRA and OpenBreast in pectoral muscle removal compared to radiologist manual segmentation. As secondary endpoint, we assessed the performance variability according to breast density and image laterality.

## Methods

### Women population

This retrospective study was approved by the ethic committee of University “Luigi Vanvitelli”, Naples, Italy, as an observational retrospective spontaneous study (Deliberation n. 469 of 23/07/2019). All methods were carried out in accordance with relevant guidelines and regulations. Informed consent was waived by the same ethic committee that approved the study.

We retrieved bilateral full-field digital mammograms (FFDMs) from consecutive women, who underwent mammography for breast cancer screening programs, at the Breast Unit of the University Hospital “Luigi Vanvitelli,” Naples, from June 2020 to November 2020. Moreover, we retrieved breast composition categories A, B, C, D (according to BI-RADS 5th edition published in 2013 [11]) that have been assessed by two expert radiologists in consensus (GG, MPB).

All examinations were performed according to the American College of Radiology (ACR) and the European Commission (EU) guidelines, [1]. We included only images satisfying quality/correctness criteria according to current best practices [1], although about 20% of images were considered not correct by the radiologist and have been repeated.

As the aim of the study was to assess pectoral muscle removal on routinely acquired images, no women inclusion/exclusion criteria were used. Breast composition has been retrieved to evaluate whether it could affect performance.

### Image characteristics

Women have been assessed in both mediolateral oblique (MLO) and craniocaudal views (CC) using the system Giotto Class produced by IMS Giotto S.p.A. (Sasso Marconi (BO), Italy).

Although both MLO and CC were available, we analyzed only MLO images due the larger presence of pectoral muscle on this kind of projection.

### Manual segmentation of pectoral muscle

According to the DICOM standard [20], mammographic images might be stored as both “For Processing” and “For Presentation.” In this study, for manual drawing purposes, “For Presentation” images, processed with algorithm "Deep View 1.0" embedded in Raffaello software by IMS Giotto S.p.A, have been used. Two expert radiologists in consensus (10 years in mammograms reviewing) have manually drawn contours of pectoral muscle on each MLO using Horos Medical image viewer v 2.0.2 [12]. A free-hand closed line has been drawn consisting of the outline of the pectoral muscle itself.

### Packages to be compared

We have compared performance of two off-the-shelf Matlab open-source packages publicly available for breast assessment: LIBRA [8] and OpenBreast [9]. These two packages differ in the detection of the pectoral muscle as briefly described in 2.4.1 and 2.4.2. Although the ultimate goal of the two packages is to provide an automatic breast density estimation, in this study we considered only data on pectoral muscle removal.

Both algorithms assume that MLO-view mammograms can be roughly subdivided into three regions: the chest wall, including the pectoral muscle; the breast tissue, comprised primarily of adipose and fibroglandular tissues; and the background air region. Thus, they perform first breast area segmentation to identify the tissue-air interface and subsequently they identify the boundary between breast tissue and the pectoral muscle.

Both packages have been run on Matlab R2017b (The MathWorks, Inc., Natik, MA, USA) [10]).

#### LIBRA

The Laboratory for Individualized Breast Radiodensity Assessment (LIBRA) [8] is an algorithm for fully automated quantification of breast percentage density (PD%) performing for both raw and processed digital mammography images. LIBRA’s breast region and pectoral muscle segmentation, which represents one of the key computational steps constituting the algorithm, employs textural features in dense-tissue segmentation. The body‐air interface boundary is determined by a threshold based on the gray‐level intensity histogram, independent of any prior assumptions. The boundary between the pectoral muscle and breast tissue areas use a previously validated algorithm based on a straight line Hough transform [13]. In this study, we used LIBRA version 1.0.4 available at the link https://www.nitrc.org/projects/cbica_libra/ provided by Perelman School of Medicine University of Pennsylvania website—SBIA participating with CBICA.

#### OpenBreast

OpenBreast [9] is a fully automatic computerized framework for mammographic image analysis implemented in three steps: breast segmentation, region of interest (ROI) detection, and feature extraction. The first phase of the breast segmentation is similar to that of LIBRA. The second phase, represented by the identification of the boundary between breast tissue and the pectoral muscle, consists of the chest wall and nipple detection, respectively. More precisely, for the detection of the chest wall a Hough-based line detector is used [14]. The line detector works by first applying an edge detector to the input image. Subsequently, each edge pixel is represented into a parametric Hough accumulator space. Lines are detected as local maxima of the space histogram. Finally, the nipple is detected as the further contour point from the chest wall [15]. OpenBreast v1.0 has been developed at Universidad Industrial de Santander, School of Electrical, Electronics and Telecommunications Engineering, Bucaramanga, Colombia, and has been downloaded at the link https://github.com/spertuz/openbreast, GitHub.

### Comparison between pectoral muscle removal

Performance of the two packages has been compared with respect to the manually segmented pectoral muscle by means of two commonly used indices for segmentation accuracy: Dice-index and Cohen-kappa [16]. Manually delineated pectoral muscle region has been considered the “ground truth” identification.

In order to perform the comparison between the segmented pectoral muscle and manually delineated pectoral muscle, we computed the number of pixels correctly identified as pectoral or breast (respectively true positive TP, true negative TN) and the number of pixels wrongly identified as pectoral or breast (respectively false positive FP, false negative FN).

Dice-index is defined as:

$$ {\text{Dice}} = {\text{2 TP}}/\left( {{\text{2 TP}} + {\text{FP}} + {\text{FN}}} \right) $$

and it measures the overlap between two segmentations [16]. However, Dice-index does not included TN and another index was used to take into account TN: the Cohen’s kappa which is defined as:

$$ {\text{kappa}} = \left( {{\text{fa}} - {\text{fc}}} \right)/\left( {{\text{N}} - {\text{fc}}} \right) $$

where N is the total number of pixels on the image and:

$$ {\text{fa}} = {\text{TP}} + {\text{TN}} $$

$$ {\text{fc}} = \left[ {\left( {{\text{TN}} + {\text{FN}}} \right)\left( {{\text{TN}} + {\text{FP}}} \right) + \left( {{\text{FP}} + {\text{TP}}} \right)\left( {{\text{FN}} + {\text{TP}}} \right)} \right]/{\text{N}}. $$

### Statistical analysis

Statistical differences between packages performance have been tested using non parametric Wilcoxon signed-rank test for paired groups; possible dependence of performance with breast density has been also assessed via Kruskal–Wallis test. Everywhere, P-values lower than 0.05 were considered significant. Given the small number of pre-planned tests, no p-value correction has been applied [26]. Statistical analysis has been performed using the R environment (https://www.R-project.org/).

## Results

### Women population and image characteristics

168 women have been included in the study, corresponding to 336 FFDM images. Women characteristics are summarized in Table 1. The detailed operative setting of mammographic image acquisition is reported in Table 2.

**Table 1:** Patients characteristics: number of patients across breast density and age group

Age group (yrs)	Breast density				
	A	B	C	D	Sum
15–44	0	6	2	7	15
45–54	4	18	25	9	56
55–64	8	41	5	15	69
65–78	5	14	0	5	24
> 78	0	3	1	0	4
Sum	17	82	33	36	168

**Table 2:** Image and equipment characteristics

Anode material	Tungsten (W)
Filter materials	0.05 mm silver (Ag); a 0.7 mm aluminum (Al) filter may be also available on the system
Detector	a-Se Flat Panel Detector
Pixel size [µm]	85
kVp	31 (26–35)
Exposure time [ms]	508 (106–1340)
mAs	75.35 (14.91–200.22)
Anode/filter combination	W/Ag
Entrance dose [mGy]	4.77 (0.87–17.31)

Table 1 reports main characteristics of our sample. Most women were between 45 and 64 years old. Moreover, a small proportion of women had A level of BI-RADS breast density. An inverse relationship between age and breast density was found (*P* < 0.05).

### Comparison between packages

**Fig. 1:**
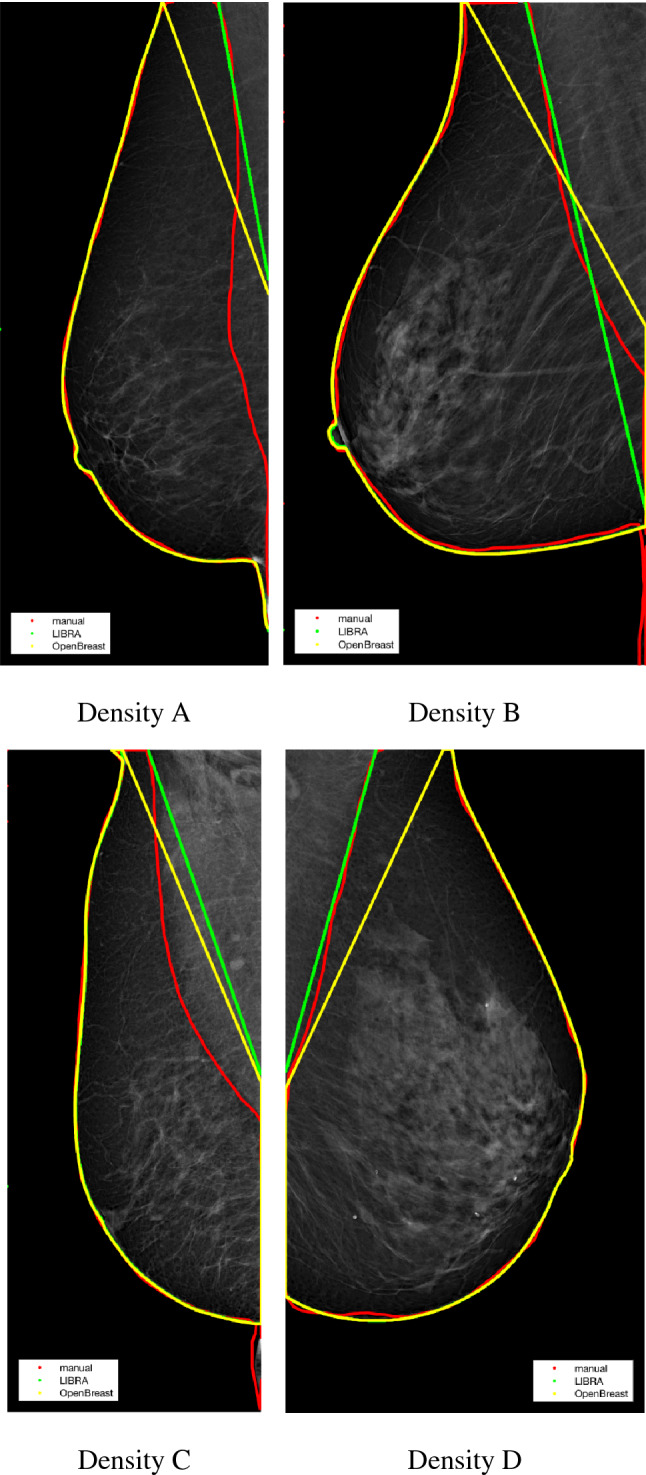
Illustrative examples of pectoral muscle delineation. Manual (red), LIBRA (green) and OpenBreast (yellow). Breast parenchyma contour is also reported for comparison. Breast density is indicated below each image

Figure 1 reports a few illustrative examples of pectoral muscle removal obtained by manual delineation (red), LIBRA (green) and OpenBreast (yellow). One example per each breast density and an example of different laterality have been reported. It can be observed that neither LIBRA nor OpenBreast can achieve very accurate approximation to the manual removal of the pectoral muscle.

**Fig. 2:**
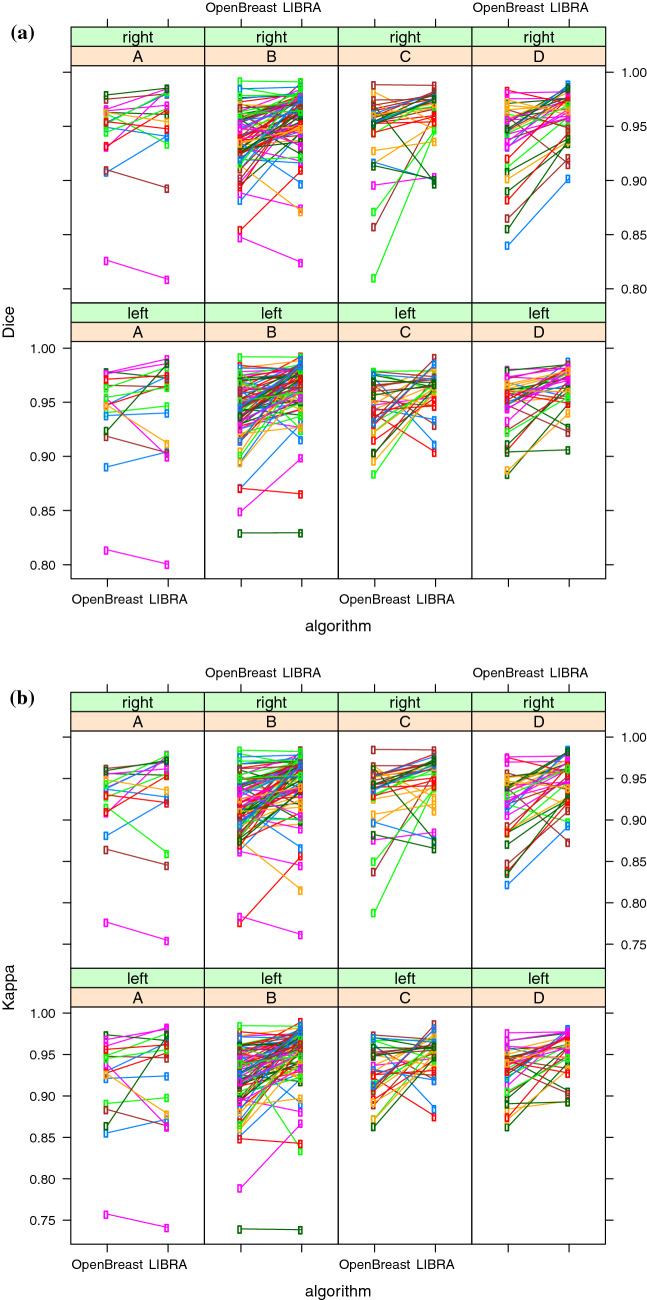
**a** Dice-index of LIBRA and OpenBreast versus gold truth has been reported per each combination of breast density (A,B,C,D) and laterality (left, right); each subject is identified by a colored circle, and the measurements on the same subject but with different packages are connected by lines. **b** Cohen’s kappa of LIBRA and OpenBreast versus gold truth has been reported per each combination of breast density (A,B,C,D) and laterality (left, right); each subject is identified by a colored circle, and the measurements on the same subject but with different packages are connected by lines

For illustrative purposes, Fig. 2a and b reports the complete distributions of Dice-index and Cohen-kappa across the sample population (336 images). The Wilcoxon signed-rank test between LIBRA and OpenBreast revealed significant differences (*P* < 0.05): both Dice and Cohen performance indices were higher on LIBRA than OpenBreast.

Table 3 reports average values of Dice-index and Cohen-kappa, and TP, TN, FP, FN fractions. Moreover, Table 3 synthesizes TP, FP, TN, FN fractions, Dice-index and Cohen-kappa across breast density type.

**Table 3:** Average Dice-index, Cohen’s kappa, TP, TN, FP, FN fractions have been reported per each package/breast density

	OpenBreast						LIBRA					
Breast density	TP	FP	FN	TN	Dice	kappa	TP	FP	FN	TN	Dice	kappa
A	0.27	0.01	0.02	0.70	0.94	0.92	0.28	0.02	0.01	0.69	0.95	0.93
B	0.25	0.01	0.02	0.72	0.94	0.92	0.26	0.01	0.01	0.72	0.96	0.95
C	0.22	0.02	0.01	0.76	0.94	0.93	0.22	0.01	0.01	0.76	0.96	0.95
D	0.24	0.02	0.01	0.73	0.94	0.92	0.24	0.01	0.01	0.74	0.96	0.95

Average values for LIBRA are higher than corresponding values for OpenBreast (*P* < 0.05). Results suggest that LIBRA can achieve better performances: in fact, both LIBRA’s Dice-index and Cohen-kappa were significantly higher than those of OpenBreast (*P* < 0.05). Average values do not depend upon breast density level. In fact, no differences statistically significance was observed among two algorithms respect to breast density value (*P* > 0.05 at Kruskal–Wallis test).

In addition, no statistically significant differences were observed among two algorithms respect to laterality (*P* > 0.05 at Wilcoxon paired test for laterality).

## Discussion

There are two issues for the detection of breast cancer at an early stage: the proper acquisition of mammogram images and, secondly, precise analysis of the images for the breast cancer diagnosis. The manual segmentation is time consuming and could delay the processing process. Accurate viewing of images is a difficult assignment, especially with a large dataset. This challenge can be overcome by using a computational method such as image processing techniques or the breast cancer analysis algorithm. These algorithms lead to rapid analysis and reduce the radiologist job of medical experts. However, it is crucial to assess the nature of mammography images before using the algorithm based on the image process to detect tumor [5–7]. The pectoral muscle, which is commonly shown in MLO viewed mammograms, is usually removed before analysis as it can be easily misclassified as fibroglandular tissues. Additionally, artifacts that are accidentally produced during image acquisition may show in pectoral muscle areas of mammography images. Moreover, pectoral muscle regions can be examined by radiologists for auxiliary lymph abnormalities.

The aim of this work was to evaluate the performance in terms of pectoral muscle removal of two open-source Matlab packages (LIBRA and OpenBreast) publicly available. The comparison was made on a single database of FFDM images from a population of consecutively assessed women. Our results indicate that LIBRA has achieved higher performance than OpenBreast in terms of both Dice-index and Cohen-kappa. No dependence of results upon breast density or laterality has been found.

The main purpose of analyzing these tools is to support the development of computerized breast cancer detection systems whose aim is to detect mammographic lesions with poor visibility: low contrast regions with small abnormalities are mostly hidden in the tissue of mammogram images, which makes it challenging to analyze the abnormal region and also provides false detection. As outlined in the introduction the task of pectoral muscle removal is an important preliminary step for computer-aided diagnosis on FFDM, because the intensity levels of pectoral muscle might affect the detection of masses, microcalcifications etc. Moreover, pectoral boundaries identification might improve and simplify breast bi-laterality evaluation tasks. As a consequence, this is still an active area of research. However, many algorithms have been proposed and no definitive results have yet been achieved, also because of the specific anatomical difficulties in delineating pectoral boundaries on FFDM.

In this context, our results provide information on the performances of two open-source packages for pectoral muscle boundary delineation in the Matlab environment. The latter is a widely used software environment for development of computer-aided diagnosis systems. Our results might allow improvements in CAD development.

As far as the authors’ knowledge, our study is the only one comparing those two packages. Moreover, in contrast to many previous studies [17–19], our study has been performed on a large database of full-field digital mammograms: at the time of writing a public database of FFDM, in the order of thousands of images, is not yet available, and many studies used databases with digitized film mammograms [20–31]. In fact, one main issue concerning breast pectoral removal is the lack of large-scale well-annotated datasets for training of high performance models. In recent years, considerable effort has been devoted to develop intelligent and robust methods for breast pectoral removal. However, the majority of the methods are evaluated on self-annotated public datasets or even private datasets due to the limited availability of datasets. To give a flavor of the type of algorithms that have been used in recent literature, we briefly describe two algorithms that have been developed using such databases. Maitra et al. [32] implemented a method based on a triangular region to isolate the pectoral muscle from the rest of the tissue. Then, a region-growing technique was used to identify and remove the pectoral muscle. Kaitouni et al. [33] implemented breast tumor identification by pectoral muscle removal based on hidden Markov and region-growing method. The purpose of the method was to separate the pectoral muscle from the mammographic image. The method involved Otsu thresholding and k-means based for pixel classification.

The need for a full-tested preprocessing tool is strong in the research community of FFDM analysis, especially as texture radiomics and deep-learning approaches have entered into the field. As a matter of fact, important issues with deep models for pectoral removal are the robustness of the methods and the training phase. Before the advent of deep learning, feature-based methods dominated the field [34–37]. The robustness of these kinds of systems remains to be improved as variations in the images could lead to wrong removal. In this sense, the advantage of deep-learning-based methods is such that the robustness has been drastically enhanced [30–32, 38–43]. However, deep-learning approach requires a very large number of images to be properly trained. Therefore, their development still remains problematic due to the lack of large FFDM databases properly manually annotated, as mentioned above. On the contrary, although not based on deep-learning approach, the two packages analyzed have been proven to be robust against various situations and turned out to be suitable for pectoral muscle removal.

This study has several limitations. First, the simple size assessed. However, the sample size analyzed in this study was justified statistically. In fact, typical values for accuracy differences among different algorithms can be expected to be a few percentage points and the expected standard deviation is form 2 to 4 times the differences [2, 3]: this yields a standardized mean difference of 0.2. Applying standard power samples size computation [4] for paired test and considering a power of 80% and alpha level of 5%, a sample size of about 150 subjects can be considered adequate. Moreover, manual accurate free-hand delineation of the pectoral muscle is a very time-consuming task which puts a fundamental limit on the size of the database which can be analyzed in a limited frame of time. Second, the population is imbalanced considering that the prevalence of women had a breast density of B type; however, no statistically significant dependence of pectoral removal performance was found respect the breast density.

## Conclusions

In summary, we compared the performance of pectoral muscle removal for the two Matlab packages LIBRA and OpenBreast on a single FFDM database with respect to manual delineation. Results indicated that both packages showed high values of agreement with manual segmentation. However, statistical tests suggest that, on average, LIBRA can achieve higher performance. Although this study has not a direct clinical application, our results are useful in the choice of packages for the development of complex systems for computer-aided breast evaluation.
